# Deriving and validating biomarkers associated with autism spectrum disorders from a large-scale resting-state database

**DOI:** 10.1038/s41598-019-45465-9

**Published:** 2019-06-21

**Authors:** Chia-Min Chen, Pinchen Yang, Ming-Ting Wu, Tzu-Chao Chuang, Teng-Yi Huang

**Affiliations:** 1 Department of Electrical Engineering, National Taiwan University of Science and Technology, Taipei, Taiwan; 2 Department of Psychiatry, College of Medicine, Kaohsiung Medical University and Kaohsiung Medical University Hospital, Kaohsiung, Taiwan; 3 Department of Radiology, Kaohsiung Veterans General Hospital, Kaohsiung, Taiwan; 4 Faculty of Medicine, School of Medicine, National Yang-Ming University, Taipei, Taiwan; 5 Institute of Clinical Medicine, School of Medicine, National Yang-Ming University, Taipei, Taiwan; 6 Department of Electrical Engineering, National Sun Yat-Sen University, Kaohsiung, Taiwan

**Keywords:** Brain imaging, Diagnostic markers

## Abstract

Resting-state functional magnetic resonance imaging (MRI) has been used to investigate the brain activity related to autism spectrum disorder (ASD). In this study, we applied information from a large-scale dataset, the Autism Brain Imaging Data Exchange (ABIDE), to clinical applications. We recruited 21 patients with ASD and 23 individuals with neurotypical development (TD). We applied ASD biomarkers derived from ABIDE datasets and subsequently investigated the relationship between the MRI biomarkers and indicators from clinical screening questionnaires, the social responsiveness scale (SRS), and the Swanson, Nolan, and Pelham Questionnaire IV. The results indicated that the biomarkers generated from the default mode and executive control networks significantly differed between the participants with ASD and TD. In particular, the biomarkers derived from the default mode network were negatively correlated with the raw scores and model factors of the SRS. In summary, this study transferred the efforts of the global autism research community to clinical applications and identified connectivity-based biomarkers in ASD.

## Introduction

Autism spectrum disorders (ASDs) are childhood developmental disorders characterized by dysfunction in social communication, interaction deficits, and specific behavioral characteristics with early onset^1^. ASDs are disorders of male preponderance, with a sex ratio of 4:1 and an estimated worldwide prevalence of 1–2.6%^2^. The developmental course, core symptoms’ severity, and intellectual and adaptive abilities can vary greatly. Impairment in social cognition is a primary feature of the clinical presentation of ASDs^3^. According to the Diagnostic and Statistical Manual of Mental Disorders, Fifth Edition (DSM-5), the main symptoms of ASD are deficits in social interaction and restricted, repetitive patterns of behavior or interests. Theory of mind (ToM) is a prominent psychological theory that explains the causes of social and emotional deficits that are the core impairments of ASD. ToM is the mentalizing system and refers to an individual’s ability to reason about other people’s beliefs or intentionality and is a crucial component of social behavior. In clinical practice, researchers use screening methods, such as the social communication questionnaire^4^ or social responsiveness scale (SRS)^5^, to assess ASD symptom severity. In particular, SRS scores help clinicians identify childhood ASDs and subthreshold ASD symptoms in children with various psychological problems.

Researchers have applied a task-free medical imaging technique, resting-state functional magnetic resonance imaging (R-fMRI), to study the brain activity underlying ToM and ASD. The instinct activity in brain regions simultaneously working together is considered to constitute brain networks, which can be measured as intrinsic functional connectivity (FC). FC analysis involves identifying the brain regions in which the blood oxygenation level-dependent signal cofluctuates. The regions can be considered one brain network for a specific cognitive ability or mental processing task. Functional MRI studies have demonstrated that ToM involves the medial prefrontal cortex (mPFC), temporoparietal junction, middle temporal gyrus (MTG), temporal pole, and posterior cingulate cortex/precuneus^6–8^. Studies using R-fMRI have reported that brain regions within the default mode network (DMN) overlapped with brain regions associated with ToM. Thus, using R-fMRI to measure FC may provide quantitative insights into the social-cognitive ability of patients with ASD^9–12^. For example, Assaf *et al*. demonstrated that FC values of specific brain regions were highly correlated with SRS scales in a group of patients with ASD. Weng *et al*. determined that FC strength between the posterior cingulate cortex and temporal lobe was negatively correlated with social impairment based on the Autism Diagnostic Interview-Revised (ADI-R). These studies have highlighted the potential applications of R-fMRI for investigating the complex cognitive function underlying ASD.

Since 2012, the Autism Brain Imaging Data Exchange (ABIDE) initiative has collected more than 2000 R-fMRI datasets of patients with ASD and individuals with neurotypical development (TD) subjects across international laboratories^13^. ABIDE could allow researchers to investigate brain mechanisms underlying ASD and to identify ASD-related biomarkers through R-fMRI^14^. This study applied information from the ABIDE initiative to investigate a local cohort. We derived R-fMRI biomarkers from ABIDE and obtained the metrics of local datasets. We analyzed and assessed the performance of the biomarkers and relationships between social responsiveness and functional brain networks in patients with ASD.

## Methods and Materials

### ABIDE: R-fMRI datasets

This study included two databases, namely ABIDE and the Kaohsiung Medical University Hospital (KMUH) databases. Table 1 lists the details. This study included 1112 ABIDE I datasets from 17 sites and 983 ABIDE II datasets from 16 sites. The ABIDE datasets were obtained online^15^. In total, 2095 ABIDE datasets were used (ASD: 1001 and TD: 1094; 5–64 years). These datasets are anonymous and in accordance with HIPPA guidelines.

**Table 1:** ABIDE and KMUH datasets.

	ABIDE		KMUH	
	ASD	TD	ASD	TD
Age (year)	15.9 ± 8.9 (5–64)	15.9 ± 8.7 (6–64)	17.1 ± 2.4 (12–21)	16.4 ± 2.7 (12–22)
Male	867	836	20	20
Female	134	258	1	3
Total	1001	1094	21	23

### ABIDE: Preprocessing and RSN10 networks

**Figure 1:**
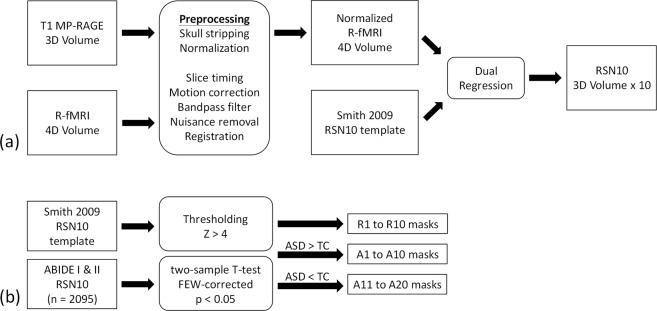
Analysis procedure for obtaining masks of the 30 biomarkers from the ABIDE datasets (**a**) producing 10 resting-state networks using a 3D T1 volume and a 4D R-fMRI volume (**b**) using a two-sample *t*-test to obtain 30 masks.

The procedure for preprocessing R-fMRI datasets and generating brain FC networks is displayed in Fig. 1(a). Anatomical 3D volumes and R-fMRI 4D volumes were processed in the FMRIB Software Library (FSL) environment. The anatomic volumes were preprocessed using FSL-BET for brain extraction and subsequently normalized to Montreal Neurological Institute (MNI) coordinates. For R-fMRI volumes, timing inconsistencies and temporal image shifts were corrected using the slice timing and image realignment functions in the FSL. Subsequently, the volumes were registered to preprocessed anatomic volumes by using FSL-BBreg and normalized to the MNI space by using the nonlinear registration tool FSL-FNIRT. The voxel size was resampled to 2 × 2 × 2 mm^3^, and the volumes were smoothed using a Gaussian filter with a full width half maximum at 6 × 6 × 6 mm^3^. The subsequent signal processing involved applying a temporal bandpass filter (0.01–0.08 Hz) to the R-fMRI volumes and regressing out 24 motion parameters obtained after the realignment procedure^16^ and five principal components with the highest variance estimated from voxel time series for the white matter and cerebrospinal fluid by using CompCor^17^. We subsequently applied dual-regression analysis^18^ by using the FSL general linear model (GLM) and the 10-brain resting-state networks (RSN10), “PNAS_Smith09_rsn10.nii.gz,” provided by Smith *et. al*.^19,20^ as a reference. The dual-regression analysis was used to assess the FC of each voxel estimated based on the GLM parameters normalized by the residual within-subject noise^18,21^. The procedure generated 10 whole-brain RSN maps for each dataset. The networks (RSN1 to RSN10) correspond respectively to the primary visual, occipital pole, lateral visual, default mode (DMN), cerebellum, sensorimotor, auditory, executive control (ECN), right frontoparietal, and left frontoparietal networks.

### ABIDE: Procedures deriving 30 biomarkers

**Figure 2:**
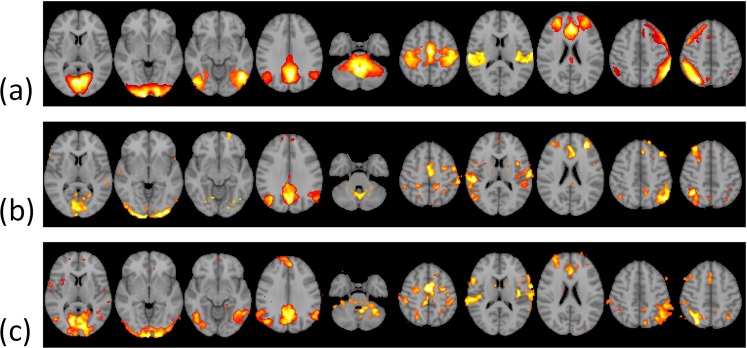
Block diagram for calculating the 30 biomarkers of individual datasets.

The block diagrams of the generation of 30 R-fMRI biomarkers, R1 to R10 and A1 to A20, are displayed in Fig. 1(b). The quantities of the biomarkers were calculated based on 30 masks. The masks for R1 to R10, termed R-masks, were generated by identifying voxels with Z values higher than 4 in the RSN10 template (PNAS_Smith09_rsn10.nii.gz) provided by Smith *et al*. (2009), and the masks for A1 to A20, termed A-masks, were created on the basis of the group difference of ABIDE RSN10 maps. Total RSN10 maps from the ABIDE datasets were 2095 (ASD: 1001 and TD: 1094) We performed a two-sample *t*-test on the ABIDE RSN10 FC maps with threshold-free cluster enhancement by using FSL-randomise with 5000 permutations for multiple comparisons. Subsequently, we identified the voxels satisfying two criteria: (1) FC values significantly different [family-wise error (FWE)-corrected *p* < 0.05] between the ASD and TD groups and (2) voxels inside the corresponding R-masks to create A1–A20 masks (ASD > TD: A1 to A10 and ASD < TD: A11 to A20). We subsequently calculated the averaged FC values of the RSN10 maps by using the masks to generate 30 biomarkers for each participant, referred to as R1 to R10 and A1 to A20 hereinafter. The procedure is illustrated in Fig. 2.

### KMUH: R-fMRI datasets

For the local cohort, 44 individuals (ASD: 21 and TD: 23; 12–22 years) were recruited from the active follow-up psychiatric clinic at KMUH and the community. Both groups of participants were between 12 and 22 years old and had scores of >70 in either the full-scale Wechsler Adult Intelligence Scale or full-scale Wechsler Intelligence Scale for Children, Fourth Edition. The participants in the ASD group were diagnosed with autistic disorder on the basis of the DSM, Fourth Edition, Text Revision symptom criteria in their early childhood in accordance with the Autism Diagnostic Observation Schedule^22^; their ASD diagnoses were confirmed using the DSM-5 before they were enrolled into this study. This study was approved by the Institutional Review Board of Kaohsiung Medical University and Kaohsiung Medical University Hospital. Informed consent was obtained from the participants’ parents and the participants themselves in accordance with the guidelines of the Institutional Committee on Clinical Investigation. The participants underwent imaging experiments performed using a 3.0 T whole-body MRI system (Siemens, Skyra, Germany), equipped with a 32-channel head coil, at Kaohsiung Veterans General Hospital. We obtained brain structural images and R-fMRI images by using a three-dimensional (3D) magnetization-prepared rapid gradient-edge (MP-RAGE) sequence and a gradient-echo echo planar imaging (EPI) sequence, respectively. The imaging parameters for 3D MP-RAGE were TR = 2000 ms, TE = 2.07 ms, FOV = 256 mm, flip angle = 9°, sagittal slices = 160, matrix size = 256 × 256, voxel size = 1 × 1 × 1 mm^3^, and TI = 900 ms. The imaging parameters for EPI were TR = 2300 ms, TE = 30 ms, FOV = 194 mm, slice thickness = 3 mm, axial slices = 40, measurements = 150, in-plane resolution = 3.03 × 3.03 mm^2^ and matrix size = 64 × 64. The total scan time of EPI was approximately 5 min.

### KMUH: Social Responsiveness Scale and Swanson, Nolan, and Pelham Questionnaire IV

The parents of the participants from KMUH completed the Chinese version of the SRS and Swanson, Nolan, and Pelham Questionnaire (SNAP-IV). The SRS is a 65-item scale that measures the severity of autism spectrum symptoms as they occur in natural social settings^5^. We obtained the Chinese version of the SRS from the developer under a license for academic use. The psychometric properties of the Chinese version of the SRS were validated by Taiwanese researchers^23^. The sum of the total raw SRS score and five subscores reflecting the factors in the model (viz., social awareness, social cognition, social communication, social motivation, and autistic mannerisms) were derived for analysis. The SNAP-IV comprises 26 items regarding the symptoms of inattention, hyperactivity/impulsivity, and oppositional defiant disorder (ODD). The Chinese version of the SNAP-IV is a reliable, valid instrument for rating the symptoms of inattention, hyperactivity/impulsivity, and ODD in both clinical and community settings. Its psychometrics properties for Taiwanese populations have been validated^24^. Three SNAP-IV scores (viz., inattention, hyperactivity/impulsivity, and ODD) for each participant were derived for analysis. Table 2 presents the average SRS and SNAP-IV scores for the KMUH datasets.

**Table 2:** Average SRS and SNAP-IV scores in the KMUH dataset.

	ASD (n = 21)	TD (n = 19)
SRS		
Total	108.95 ± 27.78	40.5 ± 22.03
Social awareness	12.14 ± 3.72	6.63 ± 2.71
Social cognition	21.43 ± 5.27	7.84 ± 4.14
Social communication	38.67 ± 10.46	11.53 ± 9.11
Social motivation	17.14 ± 5.54	9.05 ± 4.87
Autistic mannerism	19.57 ± 7.25	5.00 ± 3.73
SNAP-IV		
Hyperactivity/impulsivity	14.19 ± 6.58	6.21 ± 4.33
Inattention	7.00 ± 4.57	2.89 ± 2.11
Oppositional symptoms	6.81 ± 4.57	6.00 ± 4.22

### Statistical analysis

Total RSN10 maps from the ABIDE and KMUH datasets were 2095 (ASD: 1001 and TD: 1094) and 44 (ASD: 21 and TD: 23), respectively. We calculated the biomarkers for each RSN10 dataset and subsequently obtained two matrixes (ABIDE: 2095 × 30 and KMUH: 44 × 30) for further statistical analysis. The differences in biomarkers between the ASD and TD groups were assessed using the *t*-test. For the KMUH datasets, we performed a correlation analysis to investigate relationships between the 30 biomarkers and SRS and SNAP scores and a receiver operating characteristic analysis to evaluate the classification performances of the biomarkers.

## Results

**Figure 3:**
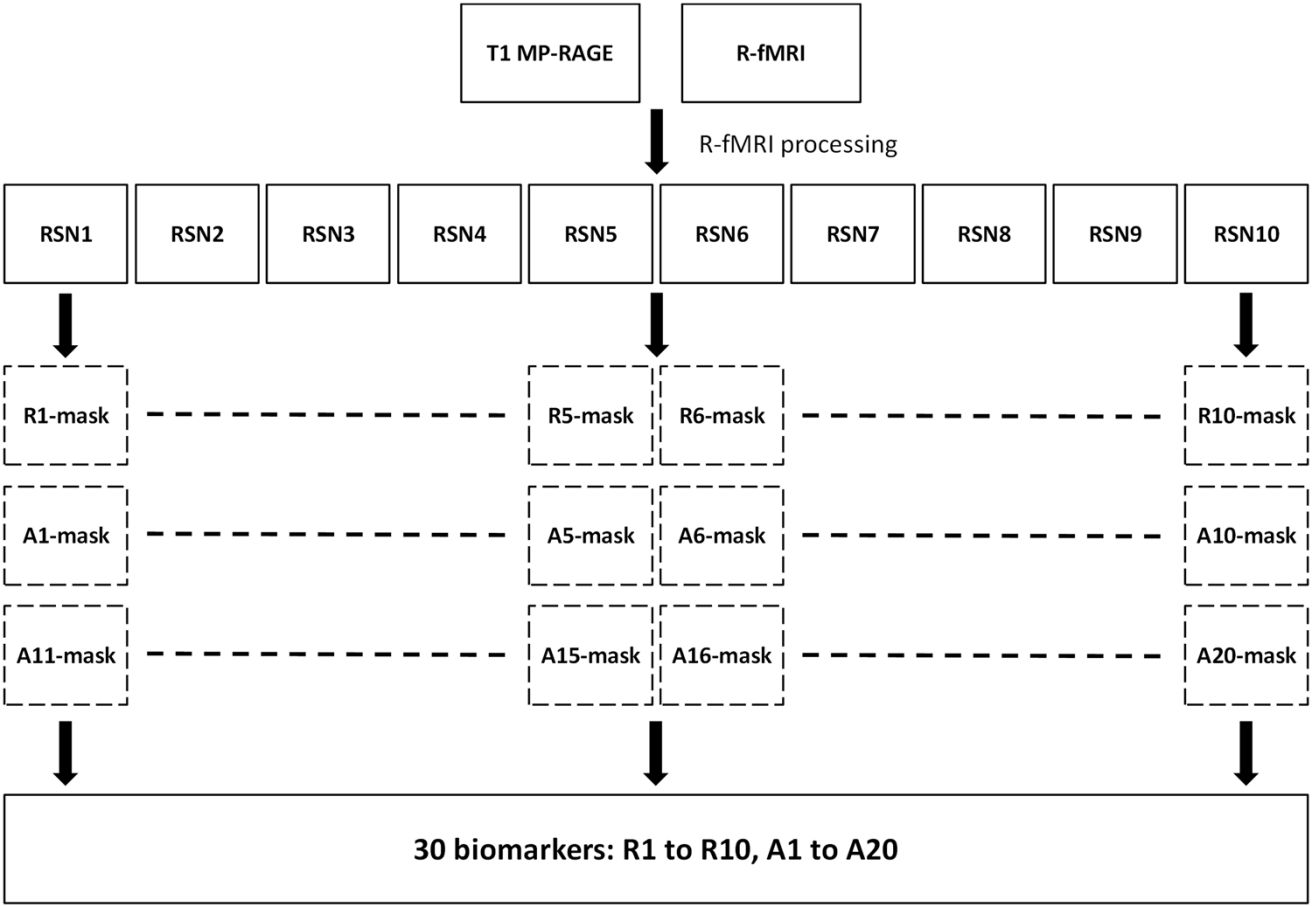
Selected slices of RSN10 templates (PNAS_Smith09_rsn10.nii.gz, Z > 4) provided by Smith *et al*. (2009) (**a**) and typical single-subject RSN10 maps (**b**) ABIDE, (**c**) KMUH, Z > 4).

**Figure 4:**
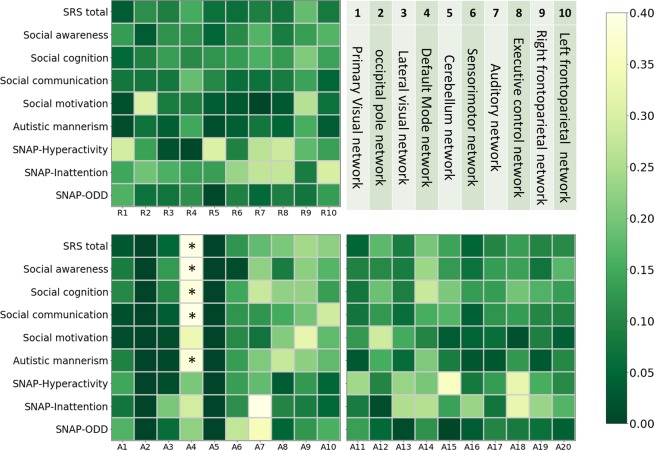
Masks of the 30 biomarkers obtained using PNAS_Smith09_rsn10.nii.gz and the ABIDE datasets.

Figure 3(a) shows the representative slices of the RSN10 templates (Z > 4) generated from PNAS_Smith09_rsn10.nii.gz. Figure 3(b,c) demonstrates typical single-subject RSN10 maps (Fig. 3b: ABIDE and Fig. 3c: KMUH; Z > 4) generated using FSL-based pipelines. Figure 4 displays the masks of the 30 biomarkers. The R-masks were produced using the RSN10 template (Z > 4), and the A-masks were derived from the group analysis of the ABIDE RSN10 FC maps (FWE-corrected *p* < 0.05, two-sample *t*-test). The volumes of the masks are listed in Table 3. The volumes of the A3, A4, and A18 masks, which ranged from 34 to 44 mL, were the top three among the A-masks. They were generated from the lateral visual, default mode, and ECN networks, respectively.

**Table 3:** The characteristics of the biomarkers in ABIDE and KMUH datasets.

Biomaker	Mask Volume (mm^3^)	ABIDE (n = 2095)		KMUH (n = 44)		
		TD (A. U.)	ASD (A. U.)	TD	ASD	AUC
R1	85704	5.36 ± 1.76	5.22 ± 1.73	5.49 ± 0.64	5.50 ± 0.89	0.518
R2	62400	4.09 ± 1.47	4.06 ± 1.40	4.38 ± 0.62	4.30 ± 0.75	0.563
R3	80048	3.79 ± 1.28	3.56 ± 1.19**	4.05 ± 0.52	3.81 ± 0.62	0.594
R4	80016	5.21 ± 1.54	4.93 ± 1.47**	4.94 ± 0.59	4.81 ± 0.65	0.590
R5	73168	3.28 ± 1.23	3.40 ± 1.26*	3.19 ± 0.72	3.88 ± 1.54	0.588
R6	75976	3.92 ± 1.41	4.05 ± 1.41*	3.72 ± 1.00	4.15 ± 1.35	0.580
R7	51952	4.17 ± 1.27	4.14 ± 1.24	3.86 ± 0.70	4.30 ± 0.97	0.642
R8	111568	3.22 ± 1.06	3.36 ± 1.09*	3.47 ± 0.88	3.97 ± 1.04	0.646
R9	99912	3.05 ± 0.89	3.08 ± 0.90	2.90 ± 0.47	3.26 ± 0.62*	0.631
R10	95304	3.49 ± 1.05	3.48 ± 1.04	3.17 ± 0.70	3.48 ± 0.68	0.640
A1	17640	5.53 ± 1.97	5.17 ± 1.90	5.59 ± 0.75	5.57 ± 1.04	0.518
A2	0	—	—	—	—	—
A3	39632	4.49 ± 1.64	4.10 ± 1.54	4.81 ± 0.68	4.45 ± 0.92	0.609
A4	34064	5.64 ± 1.68	5.18 ± 1.58	5.40 ± 0.64	4.89 ± 0.66*	0.745
A5	0	—	—	—	—	—
A6	384	3.81 ± 1.88	3.55 ± 1.81	4.37 ± 1.53	3.69 ± 1.56	0.605
A7	488	4.81 ± 1.74	4.54 ± 1.68	4.72 ± 0.91	5.13 ± 1.09	0.652
A8	11880	2.62 ± 1.03	2.34 ± 0.93	2.80 ± 0.66	2.52 ± 0.40	0.634
A9	1568	2.52 ± 1.15	2.24 ± 1.25	2.18 ± 0.95	2.28 ± 0.86	0.545
A10	1488	2.44 ± 1.36	2.14 ± 1.41	2.08 ± 0.85	1.56 ± 1.16	0.681
A11	992	2.49 ± 1.22	2.75 ± 1.32	2.12 ± 0.78	2.64 ± 1.39	0.613
A12	24	2.43 ± 2.37	2.82 ± 2.46	3.26 ± 2.18	2.53 ± 2.17	0.555
A13	1392	1.35 ± 0.90	1.57 ± 0.98	1.05 ± 0.74	1.41 ± 0.98	0.621
A14	600	2.44 ± 1.19	2.67 ± 1.29	1.92 ± 0.88	2.54 ± 1.16	0.652
A15	14704	3.64 ± 1.62	3.94 ± 1.69	3.42 ± 0.97	4.55 ± 2.04*	0.646
A16	23880	3.58 ± 1.42	3.85 ± 1.47	3.33 ± 1.09	3.98 ± 1.81	0.586
A17	1400	3.62 ± 1.55	3.87 ± 1.57	2.93 ± 1.18	3.48 ± 1.61	0.590
A18	44680	3.39 ± 1.38	3.76 ± 1.49	3.74 ± 1.17	4.71 ± 1.57*	0.677
A19	11984	1.83 ± 0.69	2.09 ± 0.77	2.11 ± 0.48	2.45 ± 0.75	0.656
A20	6616	3.31 ± 1.31	3.58 ± 1.44	3.23 ± 1.21	4.02 ± 1.53	0.687

*Significant differences between the biomarkers of TD and ASD (*p* < 0.05)

**Significant differences between the biomarkers of TD and ASD (*p* < 0.01).

Table 3 lists the mean and standard deviation of the 30 biomarkers of the ABIDE and KMUH datasets. The significance of the difference between the ASD and TD groups was assessed using the *t*-test. For the ABIDE datasets, the R-biomarkers derived from the RSN10 template (viz., R3, R4, R5, R6, and R8) provided by Smith *et al*. (2009) were significantly different between the ASD and TD groups (p < 0.05). All the 20 A-biomarkers differed significantly between the ASD and TD groups *(p* < 0.01). However, we considered the results strongly biased and excluded them from Table 3. The statistics regarding the ABIDE A-biomarkers likely overfitted because the A-masks were derived based on differences between the groups in the ABIDE datasets. For the KMUH datasets, the five biomarkers (viz., R9, A4, A14, A15, and A18) differed significantly between the ASD and TD groups (*p < *0.05, *t*-test). Of these five biomarkers, A4 and A14 were both derived from the DMN, and R9, A15, and A18 were obtained from the right frontoparietal, cerebellum, and ECN networks, respectively.

**Figure 5:**
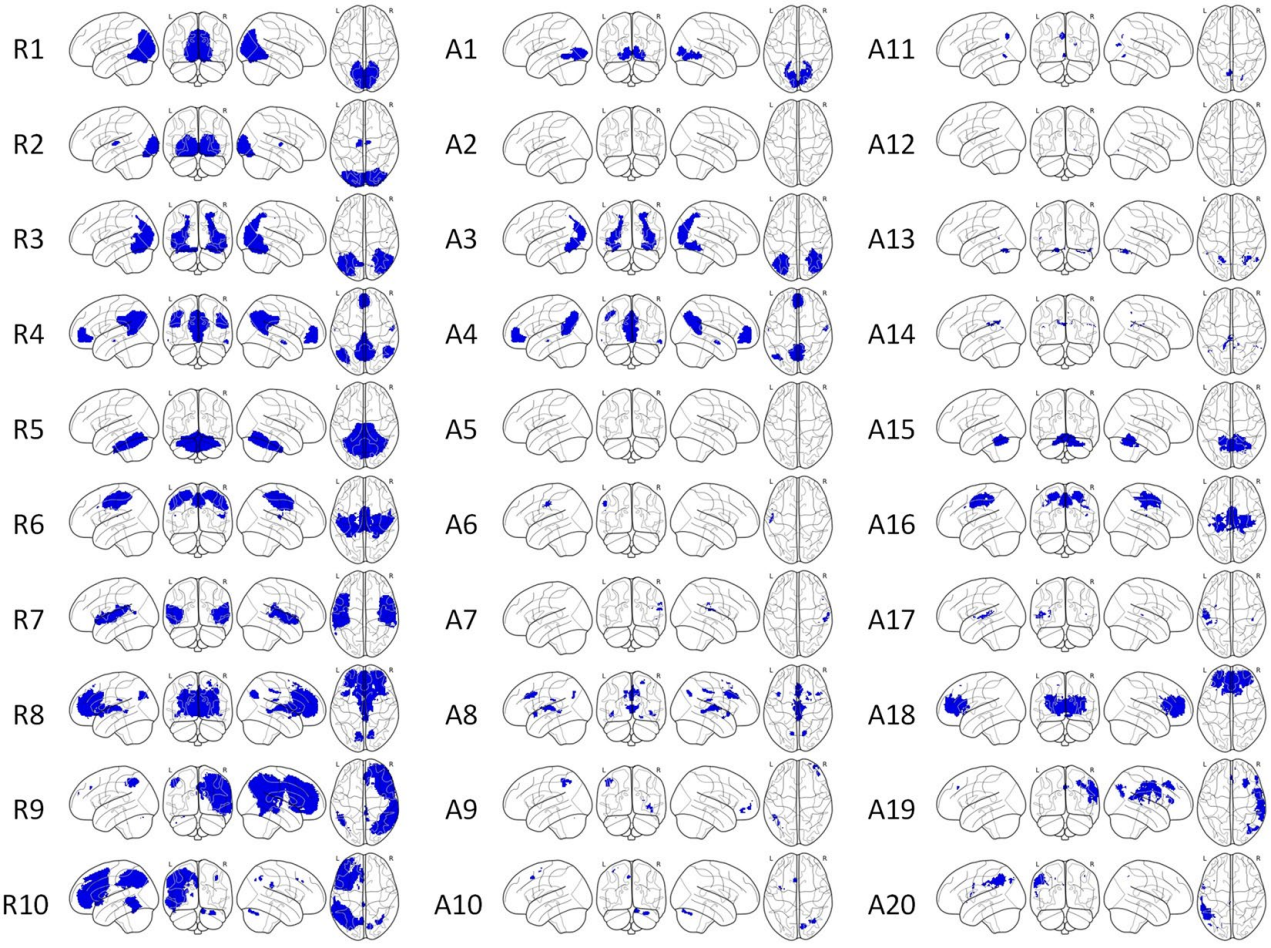
Color-coded matrix of the absolute Pearson’s correlation coefficients between 30 R-fMRI biomarkers and nine metrics of the SRS and SNAP questionnaires in the KMUH datasets (*Adjusted p < 0.05).

Table 4 lists Pearson’s correlation coefficients between the 30 R-fMRI biomarkers and the nine SRS and SNAP questionnaire metrics in the KMUH datasets. Figure 5 displays the color-coded matrix that is based on Table 4. The DMN-derived biomarker A4 (TD > ASD) and all five SRS metrics were negatively correlated (*r* = −0.333 to −0.420). In particular, the false discovery rate adjusted *p* values were statistically significant (adjusted *p* < 0.05) in five cases (viz., A4 versus SRS total, awareness, cognition, social communication, and autistic mannerism).

**Table 4:** Pearson’s correlation coefficients between R-fMRI biomarkers and metrics of questionnaires in KMUH datasets.

Biomarker	SRS total	Social awareness	Social cognition	Social com.	Social motivation	Autistic mannerism	Hyper-activity	Inattention	ODD
R1	−0.033	−0.131	0.054	−0.076	−0.017	0.011	0.292	0.144	0.162
R2	−0.114	−0.034	−0.093	−0.075	−0.306	−0.071	0.139	0.192	0.069
R3	−0.091	−0.119	−0.133	−0.079	−0.088	−0.04	0.018	−0.158	0.073
R4	−0.152	−0.12	−0.109	−0.184	−0.093	−0.146	0.005	−0.135	0.115
R5	0.068	0.049	0.121	0.106	−0.035	0.015	0.302	0.133	−0.006
R6	0.065	0.109	0.131	0.062	−0.027	0.04	0.094	0.232	0.093
R7	0.091	0.166	0.16	0.07	−0.004	0.076	0.263	0.272	0.054
R8	0.073	0.087	0.129	0.085	−0.029	0.048	0.286	0.275	0.089
R9	0.182	0.133	0.206	0.14	0.259	0.155	0.175	0.089	0.074
R10	0.082	0.167	0.141	0.051	0.069	0.035	0.135	0.298	0.13
A1	0.027	−0.088	0.105	−0.017	0.01	0.094	0.144	0.074	0.164
A2	—	—	—	—	—	—	—	—	—
A3	−0.052	−0.129	−0.107	−0.037	−0.011	−0.007	−0.012	−0.202	0.06
A4	−0.416*	−0.409*	−0.387*	−0.420*	−0.333	−0.383*	−0.203	−0.291	0.222
A5	—	—	—	—	—	—	—	—	—
A6	−0.122	−0.011	−0.145	−0.121	−0.107	−0.132	−0.133	−0.114	0.273
A7	0.181	0.223	0.28	0.122	0.072	0.207	0.181	0.422	0.349
A8	−0.204	−0.087	−0.224	−0.151	−0.21	−0.277	0.035	−0.101	0.048
A9	0.241	0.22	0.225	0.196	0.323	0.22	0.128	−0.086	0.124
A10	−0.221	−0.168	−0.133	−0.286	−0.177	−0.177	0.05	0.053	0.157
A11	0.048	−0.094	0.076	0.075	0.127	−0.03	−0.024	0.085	−0.135
A12	−0.175	−0.107	−0.188	−0.127	−0.286	−0.154	0.101	−0.011	−0.054
A13	0.087	0.086	0.09	0.064	0.151	0.059	0.23	0.252	−0.011
A14	0.199	0.233	0.281	0.155	0.101	0.205	0.201	0.259	0.118
A15	0.142	0.129	0.204	0.168	0.033	0.086	0.358	0.139	0.002
A16	0.047	0.069	0.131	0.041	−0.045	0.025	0.08	0.232	0.035
A17	0.101	0.13	0.131	0.121	0.063	0.027	0.131	0.074	−0.1
A18	0.134	0.138	0.196	0.131	0.02	0.125	0.324	0.326	0.083
A19	0.1	0.072	0.163	0.102	0.092	0.03	0.155	0.232	0.066
A20	0.109	0.171	0.132	0.094	0.039	0.106	0.107	0.166	−0.041

*Significant correlations between the biomarkers of SRS and SNAP scores (*adjusted p* < *0.05*).

Web links:

[1] http://fcon_1000.projects.nitrc.org/indi/abide/.

**Figure 6:**
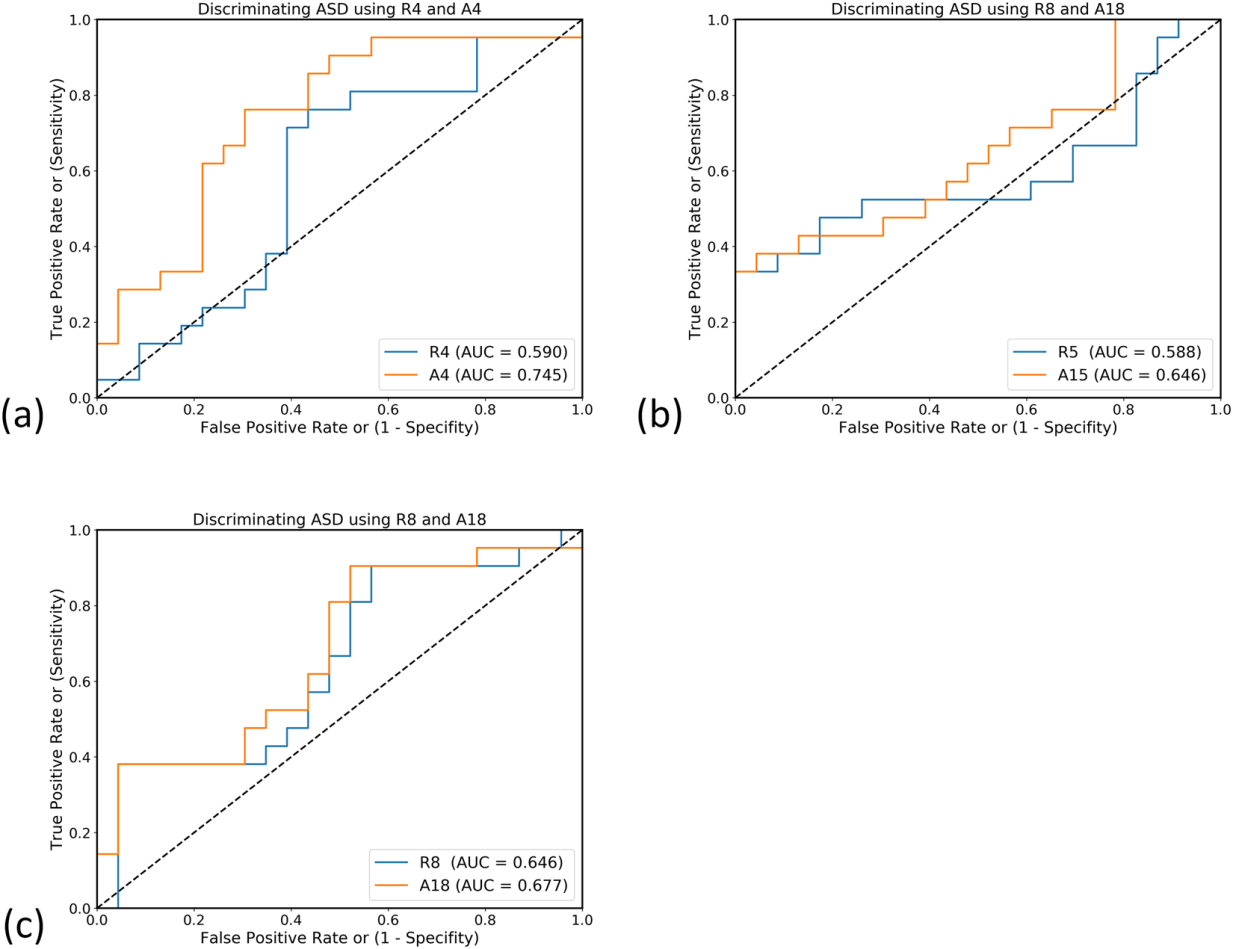
ROC curves obtained using the six biomarkers to discriminate the ASD participants from the TD individuals. (**a**) DMN: R4 versus A4 (**b**) the cerebellum network: R5 versus A5 (**c**) ECN: R8 versus A18.

**Figure 7:**
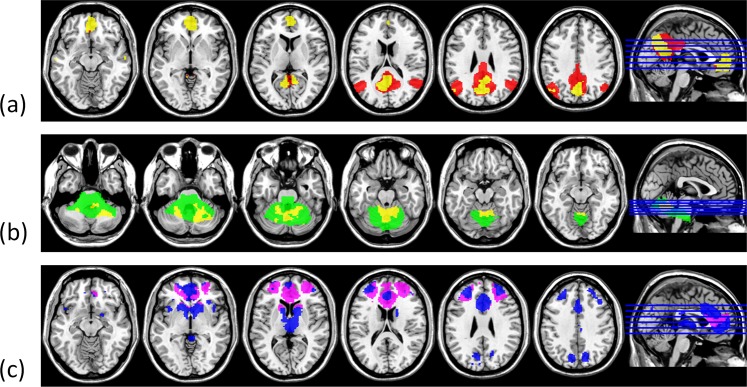
Masks of the biomarkers of the corresponding networks. (**a**) DMN (R4: red, A4: yellow), (**b**) the cerebellum network (R5: green, A5: yellow), (**c**) ECN (R8: violet, A18: blue).

A4, A15, and A18, obtained from the DMN, cerebellum, and ECN networks, respectively, exhibited a significant relationship according to the findings of difference tests and correlation analyses. We calculated the receiver operating characteristic (ROC) curves for distinguishing ASD by using the biomarkers (R4, R5, R8, A4, A15, and A18) of the three networks. Figure 6 displays the ROC curves obtained using the six biomarkers above. The areas under the curve (AUCs) for the biomarkers were (0.590, 0.745, *p* < 0.05), (0.588, 0.646), and (0.646, 0.677) for (R4, A4), (R5, A15), and (R8, A18), respectively. Figure 7 presents the masks of the three networks using different colors to highlight the R-masks and the A-masks. Although R4 and A4 were both derived from the DMN, the AUC of A4 was significantly higher than that of A8 (*p* < 0.05)^25^. The results indicate that A4 derived from the ABIDE datasets was an effective indicator for classifying ASD, and the FC of brain regions in A4 masks was correlated with cognitive impairments in patients with ASD.

## Discussion

Early in the development stage of this study, we collected R-fMRI datasets to create the KMUH cohort and explored the brain regions associated with the symptoms of ASD. We reviewed the literature, implemented pipelines to reconstruct the RSN10 maps, and used the two-sample *t*-test to evaluate the ASD and TD datasets (n = 44). The results indicated that no brain voxels were statistically significant (FWE-corrected *p* < 0.05). Meanwhile, ABIDE commenced its open-science project to provide the large-scale R-fMRI ASD database. We subsequently sought approaches to transfer ABIDE information to clinical applications involving a local cohort. We ultimately formulated an approach to extract biomarkers significantly different between ASD and TD from the ABIDE database and then validate the performance of the biomarkers using the KMUH cohort. Finally, we used correlation analysis to examine potential relationships between the biomarkers and social behaviors estimated based on clinical screening questionnaires.

We systematically analyzed the 10 RSNs of the brain in the KMUH dataset by using the RSN10 template. Although more RSNs have been reported in the literature, RSN10 networks have been consistently reported regardless of variations in acquisition protocols and analysis methods. The benefits of the analysis based on the RSN10 template are multifold. The template is publicly available; thus, researchers can compare results based on it. Smith *et al*. additionally mapped RSN10 onto behavioral domains on the basis of 7342 BrainMap activation images^26^. The mapping aided the interpretation of RSN10 components. For example, based on the behavioral mapping of RSN10, the biomarker A8 could be associated with action-inhibition, cognition, emotion, and perception–somesthesis–pain. Finally, RSN10 is now widely used in the R-fMRI research community. Although this study analyzed data using lab-made pipelines based on FSL, we found that the method for producing RSN10 maps was similar to that offered by the functions of the open source analysis project, the Configurable Pipeline for the Analysis of Connectomes (C-PAC)^27^. The pipelines, as well as the 30 masks of this study, are available^28^. The open-science materials and tools, including the RSN10 template, C-PAC, ABIDE, and our pipeline, can be used to replicate the methods of this study.

From the statistical results, we identified three sets of biomarkers that may be involved in the symptoms of ASD. They are (ABIDE, FC difference: R3, R4, R5, R6, and R8), (KMUH, FC difference: R9, A4, A14, A15, and A18), and (KMUH, FC–behavioral scores correlation A4). We observed that the three networks, DMN (R4, A4), cerebellum (R5, A15), and ECN (R8, A18), frequently presented in the three sets. The results suggest that the three networks could be the major resting-state networks associated with ASD symptoms. The ABIDE-derived features, A4 (DMN), A15 (the cerebellum network), and A18 (ECN), reached statistical significance in the difference tests in the KMUH dataset. The AUC results of A4, A15, and A18 were higher than those of R4, R5, and R8. The higher accuracy of the three A-biomarkers implied that the three R-biomarkers were not as sensitive as the three A-biomarkers used for identifying patients with ASD, and the brain regions indicated by the three masks may be the primary source of ASD.

### Default modes network: A4

The results of this study indicate that the FC of DMN in the ASD group was weaker than that of the TD group. These results are in agreement with those of previous investigations^9,29–31^. The A4 mask includes several brain regions: the mPFC, posterior cingulate cortex, left occipital cortex, and right MTG. The levels of social awareness, social cognition, social communication, social motivation, and autistic mannerisms from the SRS are all negatively correlated with the FC strength of the A4 mask. This finding is consistent with that of Assaf *et al*. who suggested that FC strength among the mPFC/anterior cingulate cortex (ACC), precuneus, and DMN correlated negatively with the SRS; in particular, weak FC strength of the ACC was correlated with higher levels of autistic mannerisms^9^.

### Cerebellum network: A15

The A15 biomarker of the cerebellum network was higher in the ASD group than in the TD group. The results suggested the cerebellum’s potential role in social-cognition behaviors. This is consistent with the findings of previous investigations. In large-scale fMRI studies on social cognition and the cerebellum, Van Overwalle *et al*. found robust clusters associated with social-cognitive studies, and their FC analysis identified the crucial role of the cerebellum in social mentalizing^32–34^. Previous imaging fMRI studies have revealed that the cerebellum activation of patients with ASD differs from that of TD individuals^35–37^. Takarae *et al*. reported that patients with ASD had increased activation of the cerebellothalamic network in a visually guided saccade experiment. Allen *et al*. identified increased and widespread activation of the cerebellum in patients with ASD compared with TD controls.

### Executive control network: A18

The ECN covers parts of the medial–frontal lobe area, including the ACC, dorsolateral prefrontal cortex, superior frontal lobe, and frontal pole^19,38^. Our results indicated that the FC values of the ECN in the ASD group were higher than those in the TD group. The derived A18 mask covers the ACC, lateral frontal gyrus, and frontal pole. The findings of the correlation analysis indicated that A18 strength was positively correlated with the levels of hyperactivity/impulsiveness and inattention behavioral problems. This network is related to several cognition paradigms, such as action-inhibition, cognition, emotion, and perception–somesthesis–pain^19^. The executive control function of attention engages more complex mental operations during monitoring and resolving the conflict between stimulus surroundings. Fan *et al*. suggested that attentional deficits contribute to the abnormalities of neuropathology in ASD and hypothesized that the attentional network system is a primary role of the pathophysiology of ASD^39^. Keehn *et al*. indicated that the orienting network was impaired in children with ASD^40^, and the orienting deficit may partly be explained by the ECN.

In summary, this study established an approach for applying information from the large-scale ABIDE database to clinical investigations of local cohorts. We obtained FC biomarkers associated with patients with ASD. They were associated with the the DMN, cerebellum network, and ECN. The results indicated that the social responsiveness of the participants was significantly correlated with the biomarkers related to the DMN.

## Data Availability

The datasets generated during and/or analyzed during the current study are available from the corresponding author on reasonable request.
